# In Silico Identification of a Potential TNF-Alpha Binder Using a Structural Similarity: A Potential Drug Repurposing Approach to the Management of Alzheimer's Disease

**DOI:** 10.1155/2024/9985719

**Published:** 2024-01-06

**Authors:** Edward Jenner Tettevi, Deryl Nii Okantey Kuevi, Balagra Kasim Sumabe, David Larbi Simpong, Mahmoud B. Maina, Julius T. Dongdem, Mike Y. Osei-Atweneboana, Augustine Ocloo

**Affiliations:** ^1^Department of Biochemistry, Cell and Molecular Biology, School of Biological Science, University of Ghana, Legon, Accra, P.O. Box LG 25, Ghana; ^2^West African Centre for Cell Biology of Infectious Pathogens, School of Biological Science, University of Ghana, Legon, Accra, P.O. Box LG 25, Ghana; ^3^Biomedical and Public Health Research Unit, Council for Scientific and Industrial Research-Water Research Institute, Accra, P.O. Box M 32, Ghana; ^4^Department of Medical Laboratory Sciences, College of Health and Allied Sciences, University of Cape Coast, Cape Coast, Ghana; ^5^Serpell Laboratory, Sussex Neuroscience, School of Life Sciences, University of Sussex, UK; ^6^Biomedical Science Research and Training Centre, College of Medical Sciences, Yobe State University, Damaturu, Nigeria; ^7^Department of Biochemistry and Molecular Medicine, School of Medicine, University for Development Studies, Tamale Campus, Ghana; ^8^CSIR-College of Science and Technology, 2nd CSIR Close, Airport Residential Area, Behind Golden Tulip Hotel, Greater Accra Region, Ghana

## Abstract

**Introduction:**

Alzheimer's disease (AD) is a neurodegenerative disorder with no conclusive remedy. *Yohimbine*, found in *Rauwolfia vomitoria*, may reduce brain inflammation by targeting tumour necrosis factor-alpha (TNF*α*), implicated in AD pathogenesis. Metoserpate, a synthetic compound, may inhibit TNF*α*. The study is aimed at assessing the potential utility of repurposing metoserpate for TNF*α* inhibition to reduce neuronal damage and inflammation in AD. The development of safe and effective treatments for AD is crucial to address the growing burden of the disease, which is projected to double over the next two decades.

**Methods:**

Our study repurposed an FDA-approved drug as TNF*α* inhibitor for AD management using structural similarity studies, molecular docking, and molecular dynamics simulations. *Yohimbine* was used as a reference compound. Molecular docking used SeeSAR, and molecular dynamics simulation used GROMACS.

**Results:**

Metoserpate was selected from 10 compounds similar to *yohimbine* based on pharmacokinetic properties and FDA approval status. Molecular docking and simulation studies showed a stable interaction between metoserpate and TNF*α* over 100 ns (100000 ps). This suggests a reliable and robust interaction between the protein and ligand, supporting the potential utility of repurposing metoserpate for TNF*α* inhibition in AD treatment.

**Conclusion:**

Our study has identified metoserpate, a previously FDA-approved antihypertensive agent, as a promising candidate for inhibiting TNF*α* in the management of AD.

## 1. Introduction

Alzheimer's disease (AD) is a progressive neurodegenerative disorder that is prevalent among the elderly population and is the leading cause of dementia [1, 2]. The prevalence of AD is increasing steadily and is predicted to double in the next 20 years [3]. Its pathogenesis encompasses the accumulation of beta-amyloid plaques, neurofibrillary tangles, and neuronal loss in the brain. It results in memory and cognitive function deterioration, which affects the daily activities of the patients [4, 5]. Despite the significant progress made in understanding the mechanism and therapeutic targets of AD, there is a lack of a definitive cure or effective treatment [6]. Consequently, AD represents a growing societal challenge and an unmet medical need [1].

Several hypotheses have been proposed over the years to explain AD's pathogenesis, with the amyloid hypothesis being the prevailing paradigm [7, 8]. However, recent studies have questioned the validity of this hypothesis and suggested alternative explanations, including the tau hypothesis, chronic inflammation, and gut microbiota theories [9, 10]. Inflammation hypothesis, in particular, postulates that proinflammatory cytokine, tumour necrosis factor-alpha (TNF*α*), plays a crucial role in AD pathogenesis. TNF*α* is upregulated in the brains of individuals with AD and impairs cognitive function [11, 12]. Additionally, studies have shown that the modulation of TNF*α* leads to variations in amyloid plaque deposition, neuronal death, and cognitive deficits, which are hallmarks of AD [13, 14]. In general, there is compelling evidence to suggest that TNF*α* plays a significant role in the pathogenesis of AD [15]. However, TNF inhibitors such as infliximab and etanercept do not cross the blood-brain barrier (BBB), which is a physical barrier that separates the brain from the peripheral circulation, limiting their efficacy in treating brain inflammation [16]. Emerging evidence suggests that the plant *Rauwolfia vomitoria* (RV) possesses compounds capable of preventing neuronal damage and reducing inflammation in the brain with minimal side effects [17–20]. This plant has exhibited promising therapeutic effects on cognitive deficit, among other plants that may have a beneficial effect on cognitive function [20–22]. *Rauwolfia vomitoria* is an ethnomedicinal plant commonly used in traditional African medicine for various ailments, including inflammation [23–25].

Plant-derived compounds have been a focal point in drug discovery for centuries, and recent advances in computational chemistry and molecular modelling have expedited the process of identifying promising drug candidates from natural sources [26, 27]. *In silico* methods have been used to predict the biological activities of plant-derived compounds, thereby speeding up the process of identifying promising drug candidates at a reduced cost [28–30]. This is particularly important for developing countries, where plant diversity is high and access to modern drug discovery technologies is limited.

Drug repurposing is the process of identifying new therapeutic uses for existing drugs. One approach to drug repurposing is based on the similarity of chemical structures between drugs.

Furthermore, drug repurposing, particularly based on structural similarity, can potentially lead to the identification of new therapeutic uses for existing drugs. Based on the idea that if two molecules share similar structures, then they may have similar bioactivities [31–34]. This approach is commonly used and aimed at identifying an analogue of an existing drug molecule that shares mechanisms of action with the original drug or compound [31, 35, 36]. Therefore, in this study, we explored the potential of *yohimbine*, the most dominant compound in the stem bark of RV, as a TNF*α* binder and potential drug candidate. Additionally, we identified an existing drug metoserpate for TNF*α* inhibition based on structural similarities.

## 2. Methods

### 2.1. Study Workflow

The study started with a systematic evaluation of the pharmacokinetic properties of *yohimbine*, the primary compound found in RV stem bark. *In silico* analysis using SwissADME was employed to comprehensively understand *yohimbine's* absorption, distribution, metabolism, and excretion (ADME) profile. Subsequently, molecular docking of *yohimbine* and the TNF*α* receptor was performed using SeeSAR software to determine their binding affinity. A structure similarity search for *yohimbine* was conducted to identify compounds with a similarity of at least 75%. The pharmacokinetic profile of the selected compound was evaluated using SwissADME. The most promising FDA-approved drug was chosen based on its pharmacokinetic properties and its ability to bind to the TNF*α* receptor. The selected drug underwent docking and molecular dynamics simulations using GROMACS software to assess its stability and potential *in vivo* performance.

### 2.2. Retrieval and Preparation of 3D Protein Structure

The three-dimensional (3D) conformation of TNF*α* (PDB ID: 2AZ5; X-ray diffraction resolution: 2.10 Å), as previously reported by He et al. [37], was obtained from the Research Collaboratory for Structural Bioinformatics Protein Data Bank (RCSB PDB) [38] (https://www.rcsb.org). The retrieved protein structure was subjected to preparation using the Biovia Discovery Studio Visualizer v2021 [39]. During protein preparation, all multiple chains were eliminated from the structure, resulting in the retention of chain “**A**” for subsequent molecular docking. Additionally, the water molecules and heteroatoms that were irrelevant to the investigation were removed during the protein preparation process.

### 2.3. Retrieval and Preparation of 3D Conformer Compounds

The dominant compound, specifically *yohimbine*, in the stem bark of RV, was analysed in terms of their 3D conformer structure, as obtained from the PubChem database [40] (https://pubchem.ncbi.nlm.nih.gov/). The structure of *yohimbine* was processed using Avogadro v1.2.0 [41] (https://avogadro.cc) with the MMFF96 force field applied for the minimisation of the ligand after the addition of hydrogen atoms and the refinement of the geometry.

### 2.4. Pharmacokinetic Assessment of *Yohimbine*

In this study, a comprehensive analysis of the ADME (absorption, distribution, metabolism, and excretion) profile of the compounds was performed. Compliance with the Lipinski rule of 5 [42], which includes the parameters of molecular weight (MW), lipophilicity (log *P*), hydrogen bond acceptor (HBA), and hydrogen bond donor (HBD), was evaluated. Additionally, the GI absorption and penetration of the BBB of the compounds were examined using various models, Ghose's rule [43], Egan's rule [44], Muegge's rule [45], and Veber's rule [46]. The Ghose rule defines acceptable compounds as having a molecular weight between 160 and 480 g/mol, a log *P* value between -0.4 and 5.6, the number of hydrogen bond donors less than or equal to 5, and the number of hydrogen bond acceptors less than or equal to 10. Egan's rule considers molecular weight, log *P*, the number of hydrogen bond donors, the number of hydrogen bond acceptors, and the number of rotatable bonds, while Veber's rule takes into account the number of rotatable bonds, the number of hydrogen bond donors, the number of hydrogen bond acceptors, and molecular weight. Muegge's rule assesses the acceptability of compounds based on their molecular weight, the number of hydrogen bond donors, the number of hydrogen bond acceptors, and the topological polar surface area (TPSA). The SwissADME open-access online tool was employed to evaluate the ADME profile of the compounds assessed in this study [47] (http://www.swissadme.ch).

### 2.5. Molecular Docking of TNF-Alpha, *Yohimbine*, and Metoserpate Using SeeSAR

Molecular docking simulations were carried out using the SeeSAR module in BioSolveIT, following the default parameters. To generate the receptor grid, the AutoGrid tool in SeeSAR was used and placed at the active site of the receptor protein (Cys69, Lys98, Ser99, Pro100, Cys101, Gln102, Arg103, Glu104, Thr105, Trp114, Tyr115, Glu116, and Pro117). The ligand was then docked into the receptor utilizing SeeSAR's standard precision (SP) mode. Finally, the top-ranking poses were analysed using the Pose Viewer tool integrated within SeeSAR [48] (https://www.biosolveit.de).

### 2.6. Structural Similarity Search of DrugBank Compounds

In this study, we used the DrugBank and SwissSimilarity tool [49] to investigate drug structural similarity using *yohimbine* structure as a query. Specifically, we used SwissSimilarity, which is an open-access web-based tool that allows molecular structure comparisons of drugs based on their chemical properties. The similarity search was performed against the DrugBank database [50], which provides comprehensive data on the chemical structure, pharmacology, and clinical applications of drugs. Notably, we opted for the 2D and 3D combined DrugBank option of the SwissSimilarity web platform for the search of structurally similar drugs, employing a similarity threshold of 75% and above. Equally, the chemical structure search feature was used for the *yohimbine*-centered approach to investigate drugs similar to *yohimbine*.

### 2.7. Pharmacokinetic Assessment of Identified DrugBank Compounds

Pharmacokinetic assessment was carried out for identified drugs with structural similarity equal to or greater than 75% by evaluating the gastrointestinal (GI) absorption and BBB penetration for the selected drugs using the SwissADME web tool [47] (http://www.swissadme.ch) with focus on two important aspects (GI and BBB) of drug distribution in the body.

### 2.8. Molecular Dynamics Simulation of the TNF*α*-Ligand Complex

Molecular dynamics (MD) simulations were undertaken using the GROMACS package [51, 52] (https://www.gromacs.org) within the myPresto portal v5 software, using default force field settings (AMBER ff99SB, TIP3P, and GAFF ver2.1) [53, 54]. The entire MD process was carried out using the autodynamics options for 100 nanoseconds (100 ns (1000 ps)) [55, 56]. The MD simulation was performed on TNF*α*-metoserpate and TNF*α*-cocrystallized ligand (small molecule (C_32_ H_32_ F_3_ N_3_ O_2_)) complexes.

## 3. Results and Discussion

### 3.1. Pharmacokinetics of *Yohimbine*

The study of the pharmacokinetics of potentially therapeutic compounds is of clinical importance in the drug development process. Elsewhere, about 40% of drug candidates do not pass the clinical trial stages [57] due to undesired absorption, distribution, metabolism, and excretion (ADME) profiles of the drug candidates. For a compound to be considered a good candidate depends on its exposure to the molecular target, which is determined by absorption and metabolism and particularly for central nervous system (CNS) drugs, an ability to cross the BBB [58]. From Table 1, it can be inferred that *yohimbine* demonstrated high GI absorption and lipophilicity making it easier to cross the blood-brain barrier. A few pharmacokinetic principles pioneered by Lipinski, Ghose, Veber, Egan, and Muegge were applied to assess the drug-likeness of the plant compound *yohimbine*. *Yohimbine* was subjected to Lipinski's rule of 5, per the rule; orally active drugs should not violate any of these four criteria: molecular weight ≤ 500, log *P* (lipophilicity) ≤ 5, number of hydrogen bond donors ≤ 5, and number of hydrogen bond acceptors ≤ 10 [42]. Based on the physicochemical properties of *yohimbine*, none of the rules were violated (Table 1); this confers its use as an oral pharmaceutical drug. The total polar surface area (TPSA) for *yohimbine* was 65.56 Å^2^ which is less than 140 Å^2^ indicating good permeability in cellular lipid membranes according to Veber's rule [46]. It is evident in literature that there is a strong correlation between high TPSA and low blood-brain penetration [59–61]. The Ghose filter was applied to evaluate the drug-likeness of *yohimbine*; again, no rule was violated. Egan and Muegge's filters were employed to assess the oral bioavailability based on the physicochemical properties; once more, *yohimbine* was compliant with all the rules [62].

### 3.2. Molecular Docking of TNF*α*-*Yohimbine*

**Figure 1:**
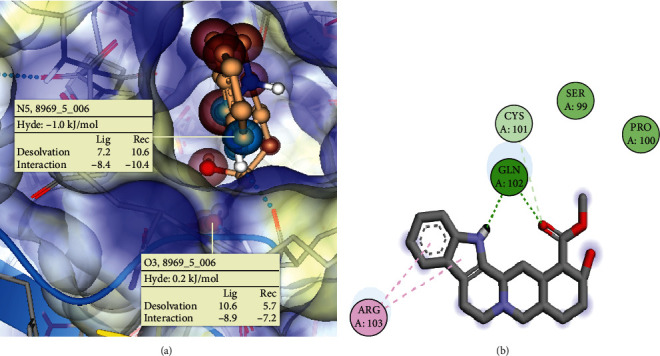
(a) A 3D representation of the complex formed between TNF*α* and *yohimbine* using SeeSAR software. The Hyde score indicates the binding affinity of the ligand to the protein. The ligand's N5 and O3 atoms form hydrogen bonds with the protein's Gln102 residue, contributing to the stability of the complex. (b) Molecular docking of *yohimbine* with TNF*α* protein. 3D structure of the protein-ligand complex visualized using Biovia Discovery Studio. The ligand (in grey, red, and purple) forms a hydrogen bond network with Gln102 (in green dashed lines) through its N5 and O3 atoms and a nonconventional hydrogen bond network between the O3 atom of *yohimbine* and Cys101 (in light green dashed lines). The ligand also interacts with Arg103 through a pi-alkyl interaction (mauve dashed line). The ligand is surrounded by a hydrophobic contact area (in light blue shade) involving Gln102 and Arg103.

The molecular docking result obtained between TNF*α* and *yohimbine* showed that there was a binding affinity Hyde score of -1.0 kJ/mol between the nitrogen atom at position 5 of the ligand and the amino acid residue Gln102 of TNF*α* (Figure 1(a) and Table 2). Additionally, there was another bond interaction (Hyde: 0.2 kJ/mol) between the oxygen atom at position 3 of the ligand and the amino acid residue Gln102. The observation of a binding affinity Hyde score of -1.0 kJ/mol between the nitrogen atom at position 5 of *yohimbine* and the amino acid residue Gln102 of TNF*α* suggests that *yohimbine* may bind to TNF*α*'s active site and inhibit its proinflammatory effects. Additionally, the bond interaction between the oxygen atom at position 3 of *yohimbine* and Gln102 may contribute to the overall stability of the *yohimbine*-TNF*α* complex. Upon analysing the docking pose using Biovia Discovery Studio Visualizer, it was observed that the ligand formed two conventional hydrogen bond networks with the amino acid residue Gln102 of TNF*α* (Figure 1(b)). In addition to the conventional hydrogen bond networks, a nonconventional hydrogen bond network was also detected between the ligand and the amino acid residue Cys101 of TNF*α*. Furthermore, two pi-alkyl bond network interactions were observed between the ligand and the amino acid residue Arg103 of TNF*α* (Figure 1(b)). Hydrophobic interaction was also observed between *yohimbine* and the TNF*α* residues Arg103 and Gln102. These results suggest that *yohimbine* has potential to bind to TNF*α* at its binding site and inhibit its proinflammatory effects.

From our study, *yohimbine*, an alkaloid with purported aphrodisiac properties and used for treating erectile dysfunction [63, 64], has been identified as a potential inhibitor of TNF*α*, a cytokine that mediates inflammation in the central nervous system (CNS) and causes oxidative stress, apoptosis, and synaptic dysfunction in neurons [65]. Neuroinflammation and resultant neurodegeneration can be precipitated by activated microglia, the resident immune cells of the CNS [66, 67]. Therefore, identifying small molecules capable of inhibiting TNF*α* could be therapeutically beneficial in treating neurodegenerative disorders associated with chronic inflammation. Here, we utilized SeeSAR, a structure-based drug design software tool, to study the interaction between *yohimbine* and TNF*α* [68, 69]. Our analysis has demonstrated that *yohimbine* exhibits a stable interaction with TNF*α*, as indicated by a Hyde score of -1.0 kJ/mol, suggesting favourable binding. Further examination of the molecular interactions has revealed key findings. Notably, a pi-alkyl bond network is formed between *yohimbine* and the amino acid residue arginine at position 103 (Arg103) of TNF*α*. Additionally, a conventional hydrogen bond is observed between the hydrogen of the imine functional group of *yohimbine* and the amino acid residue glutamine at position 102 (Glu102) of TNF*α*. Furthermore, a nonconventional hydrogen bond network interaction between *yohimbine* and TNF*α* is observed at the amino acid residue position Cys101, which contributes significantly to the binding process. These molecular interactions, as illustrated in Figures 1(a) and 1(b), play a prominent role in driving the binding between *yohimbine* and TNF*α*.

These findings provide valuable insights into the specific mechanisms underlying the interaction between *yohimbine* and TNF*α*, shedding light on the potential efficacy of *yohimbine* in modulating TNF*α* and its implications for addressing the pathogenesis of AD. Therefore, we posit that *yohimbine* may act as an inhibitor of TNF*α* and reduce its proinflammatory and neurotoxic effects in the CNS, which could explain the benefit reported for cognitive impairment and motor dysfunction [70, 71].

Compared to other TNF*α* inhibitors like etanercept and infliximab, which are large molecules and have difficulty penetrating the blood-brain barrier and pose systemic safety concerns [72–74], *yohimbine* is a small molecule that can easily cross the blood-brain barrier and has a relatively good safety profile when used at low doses [75]. Therefore, we propose *yohimbine* as a model molecule for the repurposing of an old FDA-approved drug (that may have superior bioavailability and safety profiles) for a new drug target (TNF*α*) inhibition, based on structure similarity search.

### 3.3. Identified Structurally Similar DrugBank Compounds

The primary objective of this study is to explore structure-based drug design strategies in order to identify and repurpose known compounds, like *yohimbine*, for potential therapeutic use for the management of AD. To this end, we conducted a search of the DrugBank database for FDA-approved compounds that exhibited a high percentage structural similarity to *yohimbine*. From our analysis, a total of 10 compounds with a structural similarity of at least 75% to *yohimbine* were retrieved (Table 3). These compounds include metoserpate, deserpidine, 18-methoxycoronaridine, CP-320626, rescinnamine, reserpine, raubasine, methoserpidine, (7as,12ar,12bs)-1,2,3,4,7a,12,12a,12b-Octahydroindolo[2,3-a]Quinolizin-7(6h)-One, and vinburnine. After assessing the retrieved compounds for their current FDA approval status, four of the entries were found to have FDA approval.

Metoserpate (DB11530) demonstrated the highest percentage structure similarity (0.992%) to *yohimbine*, ability to traverse the BBB, high GI absorption, and preexisting approval for clinical use, thus making it an ideal candidate for further investigation (Tables 3 and 4). The observed high degree of similarity between metoserpate and *yohimbine* can be attributed to the presence of a pentacyclic yohimban skeleton, involving the formation of a carbocyclic ring from the C-17 to C-18 bond in a corynantheine precursor, as previously reported [63].

### 3.4. Pharmacokinetics of the Identified Structurally Similar DrugBank Compounds

We assessed the GI absorption and the capacity to cross the BBB of the 10 compounds retrieved from the DrugBank database. Our findings showed that all 10 compounds had high GI absorption, indicating that they are likely to be well absorbed in the gastrointestinal tract (Table 4). However, only five of the compounds had the capacity to cross the BBB (Table 4), indicating that they may have potential therapeutic applications for the treatment of CNS disorders. These five compounds may be able to penetrate the BBB due to their physicochemical properties, such as their lipophilicity and molecular weight.

Further analysis revealed that out of the five compounds that are able to cross the BBB, only one (metoserpate) had FDA approval. Thus, metoserpate (DB11530) was the ideal candidate not only because it is the only FDA-approved drug, but also it exhibited high gastrointestinal absorption and a propensity to cross or permeate the blood-brain barrier. Metoserpate has a total polar surface area (TPSA) of 73.02 Å^2^ contributing to its ability to permeate cellular membranes. It is evident in literature that TPSA values less than 73.02 Å^2^ are indicative of good permeability and satisfy Veber's rule [46]. Metoserpate was thus selected for further analysis.

### 3.5. Molecular Docking of TNF-Alpha and Metoserpate

**Figure 2:**
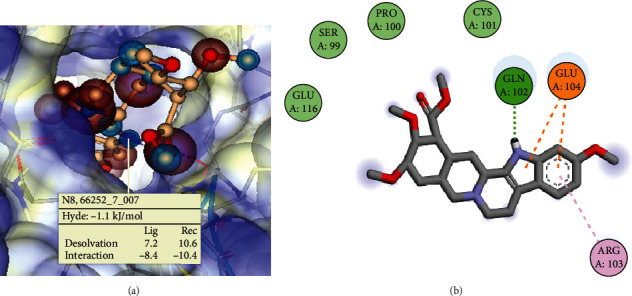
(a) A 3D representation of the complex formed between TNF*α* and metoserpate using SeeSAR software. The Hyde score indicates the binding affinity of the ligand to the protein. The ligand's N8 atom form hydrogen bonds with the protein's Gln102 residue, contributing to the stability of the complex. (b) Molecular interactions of TNF*α*-metoserpate complex. 3D visualization of the complex using Biovia Discovery Studio. The ligand (in grey, red, and purple) forms a hydrogen bond network with Gln102 through its N8 atom (in green dashed lines). The ligand also interacts with Arg103 through a pi-alkyl interaction (in mauve dashed line). A hydrophobic contact area (in light blue shade) is observed between the ligand and residues Gln102 and Glu104 (in light blue shade). Cation-pi interactions (in golden yellow) are formed between metoserpate and Glu104.

The binding affinity of TNF*α* and metoserpate was assessed using Hyde's score method. This method seeks to address weak or questionable hydrogen bonds as well as indifferent scaffolds not contributing to the free energy in the protein-ligand complex [76, 77]. From Figure 2(a), it can be observed that the Hyde score was -1.1 kg/mol which confers a favourable interaction [77]. The docking analysis revealed one hydrogen bond between the nitrogen atom at position 8 of metoserpate and the amino acid residue Gln102 of TNF*α*. These results suggest that the interaction between metoserpate and TNF*α* at this site may have potential therapeutic implications for the treatment of TNF*α*-related diseases (Figure 2(a) and Table 2).

When the docking simulation result was visualized using Biovia Discovery Studio Visualizer, one pi-alkyl bond network between metoserpate and TNF*α* binding site amino acid (AA) residue Arg103 and two salt bridge interactions between metoserpate and the binding site AA residue Glu104 of TNF*α* were observed. In addition, one conventional hydrogen bond network was observed between metoserpate and TNF*α* binding site residue Gln102 (Figure 2(b)). It is documented that conventional hydrogen bonds aid in the stability of complexes, hence conferring a good binding affinity [78, 79]. Consequently, the glutamic acid (Glu104) of the protein participated in two cation-pi interactions between the imine functional group and the benzene ring of metoserpate is shown in yellow. Cation-pi interactions play an important role in determining protein structure as well as contributing significantly to the binding energy of the complex formation [80]. Arginine (Arg103) of the protein residue participated in a pi-alkyl interaction with the benzene ring of our target drug metoserpate. According to literature, pi-alkyl interactions have a greater propensity for stability when compared to alkyls bound to nonaromatic moieties in a ligand [81–83].

### 3.6. Molecular Dynamics Simulations

#### 3.6.1. TNF*α*-Small Molecule and TNF*α*-Metoserpate

**Figure 3:**
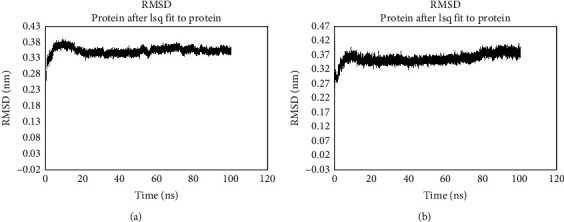
Trajectories of the overall RMSD: (a) TNF*α*-small molecule complex; (b) TNF*α*-metoserpate complex. RMSD of the various complexes with respect to the starting structure over 100 ns MD simulation. The *x*-axis represents the simulation time in nanoseconds. The *y*-axis represents RMSD in nanometers.

Numerous significant pharmaceuticals and hundreds of natural products with promising bioactivities contain indole alkaloids or have structures that are like indole alkaloids. Despite not always adhering to Lipinski's rules, such compounds frequently exhibit favourable pharmacokinetic profiles with respect to cyclic molecules. The values of the root mean square deviation (RMSD) affirm whether a close-match docked pose was predicted between the crystal and the predicted structures. It is evident in literature that an RMSD value ≤ 0.2 nm is fairly good [84–86]. Figures 3(a) and 3(b) highlight the results of TNF*α* and the cocrystallized small molecule and TNF*α* and the target drug metoserpate both having their RMSD value ≤ 2 Å (0.2 nm) which confers a latent stable protein-ligand complex.

The RMSD between the TNF*α*-small molecule complex and the TNF*α*-metoserpate complex remained consistent throughout a 100 ns simulation. However, when comparing the TNF*α*-small molecule complex (Figure 3(a)) to the TNF*α*-metoserpate complex (Figure 3(b)), a more stable trajectory was observed in the TNF*α*-metoserpate complex. In the case of the TNF*α*-small molecule complex, it displayed stability from 20 ns to approximately 30 ns, followed by a deviation. It then regained stability until around 55 ns but experienced another deviation until 60 ns. From this point, it became stable again until approximately 75 ns, with another observed deviation until around 82 ns. Finally, it regained stability and remained stable until the end of the simulation at 100 ns. On the other hand, the trajectory of the TNF*α*-metoserpate complex showed stability from around 15 ns to approximately 70 ns, with a slight deviation occurring until 80 ns. After this point, it regained stability and remained stable until the end of the simulation at 100 ns. Both complexes exhibited deviations within a range of 0.05 nm.

The observation of stable RMSD values throughout a 100 ns simulation suggests that the overall conformation of the TNF*α*-small molecule complex and TNF*α*-metoserpate complex remained relatively consistent during the simulation period [87]. This stability is an important characteristic as it indicates that the complexes maintained their structural integrity and did not undergo significant conformational changes. TNF*α*-metoserpate complex exhibited a more stable trajectory compared to the TNF*α*-small molecule complex suggesting that the binding of metoserpate, a small compound, may have induced more favourable interactions and a more stable complex formation. This could be attributed to specific molecular interactions, such as hydrogen bonding, electrostatic interactions, or hydrophobic interactions between metoserpate and TNF*α*. These interactions may contribute to a stronger binding affinity and a more stable conformation for the TNF*α*-metoserpate complex [88].

**Figure 4:**
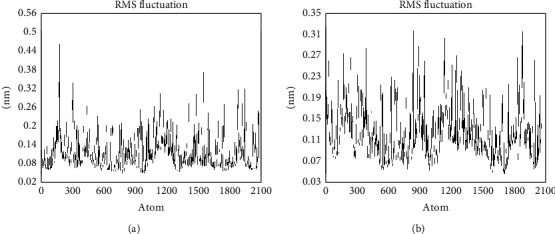
Residue-wise RMSF profiles of the TNF*α* and various ligand complexes: (a) TNF*α*-small molecule complex; (b) TNF*α*-metoserpate complex. The *x*-axis represents the atom number. The *y*-axis represents RMSF in nanometers.

To describe the local conformational change in the TNF*α* and metoserpate and TNF*α*-small molecule complexes, the root mean square fluctuation (RMSF) was required. Figures 4(a) and 4(b) highlight the RMSF profile of the TNF*α*-small molecule and TNF*α*-metoserpate complexes, respectively. From the graph, stable fluctuations were observed with RMSF ≤ 0.2 nm in both instances [89]. The TNF*α*-small molecule complex (Figure 4(a)) and TNF*α*-metoserpate complex (Figure 4(b)) both displayed reasonably low RMSF. However, the TNF*α*-metoserpate complex exhibited slightly higher fluctuations compared to the TNF*α*-small molecule complex. It is important to note that all the observed fluctuations in the TNF*α*-metoserpate complex were generally around 0.2 nm. On the other hand, in the TNF*α*-small molecule complex, fluctuations around atom positions 180 and 1520 were observed to be around 0.3 nm.

These fluctuations, measured in nanometers, indicate the degree of movement or flexibility of specific atoms within the complexes. The relatively low RMSF values suggest that overall, the complexes remained relatively stable during the simulation [90]. However, the slightly higher fluctuations in the TNF*α*-metoserpate complex could imply that the binding of metoserpate induced some additional dynamics or flexibility in certain regions of the complex compared to the TNF*α*-small molecule complex [90]. The specific atom positions 180 and 1520 in the TNF*α*-small molecule complex experienced slightly higher fluctuations around 0.3 nm. These positions could correspond to specific residues or functional regions within the complex. The increased fluctuation at these positions may indicate potential conformational changes or greater flexibility in those regions, possibly influenced by the presence of the small compound or specific interactions between the compound and TNF*α* [90].

**Figure 5:**
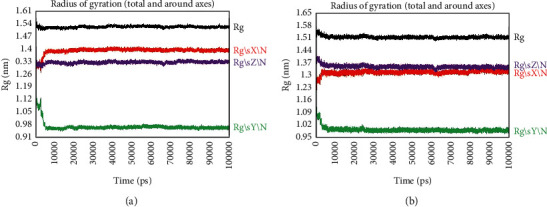
Radius of gyration profiles of the TNF*α* and various ligand complexes: (a) TNF*α*-small molecule complex; (b) TNF*α*-metoserpate complex. The *x*-axis represents the time in picoseconds. The *y*-axis represents Rg in nanometers.

The radius of gyration (Rg) monitors the compactness of the protein structure coupled with the binding patterns of the drug and protein in direct relation to the folding rate [91]. A conformational change occurs when a ligand or lead molecule attaches to the protein, changing the radius of gyration [92]. The TNF*α*-small molecule complex (Figure 5(a)) and TNF*α*-metoserpate complex (Figure 5(b)) exhibited similar total radius of gyration values, both measuring approximately 1.52 nm. A smaller radius of gyration indicates a more compact and tightly packed structure, while a larger radius of gyration suggests a more extended or flexible conformation [90]. The fact that both the TNF*α*-small molecule complex and TNF*α*-metoserpate complex demonstrated a total radius of gyration around 1.52 nm suggests that they possess comparable overall compactness, indicating a compact and stable conformation [93]. This similarity in size could indicate that the binding of both the small molecule and metoserpate did not significantly alter the overall conformation or compactness of the TNF*α* complex.

#### 3.6.2. Bond Network Evaluation of Metoserpate and TNF-Alpha Complex following Molecular Dynamics Simulation

**Figure 6:**
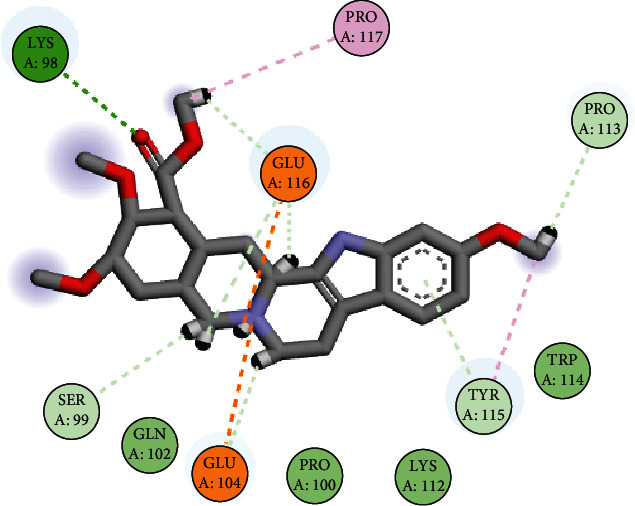
Molecular interactions between TNF*α* and metoserpate after molecular dynamics simulation. The protein residues involved in binding are represented in disc shape with different colours. The ligand is shown in ball-and-stick representation with different colours for different atom types. The molecular interactions are depicted by dashed lines with different colours indicating different types of interactions: conventional hydrogen bonds (deep green), nonconventional hydrogen bonds (light green), pi-alkyl interactions (mauve), and cation-pi interactions (golden yellow).

The post-MD simulation analysis revealed significant changes in the metoserpate-TNF*α* complex compared to the pre-MD simulation complex. Our findings demonstrated that metoserpate established multiple bond network interactions with the AAs in the binding site of TNF*α*. Specifically, a conventional hydrogen bond (cH-bond) was formed between the oxygen of the carboxylic acid methyl ester of metoserpate and the amino acid residue Lys98 of TNF*α*. Conventional hydrogen bonds are known for their strength and contribute to strong binding affinity. Additionally, several nonconventional hydrogen bonds (ncH-bonds) were observed between metoserpate and the AAs Ser99, Glu104, Pro113, Tyr115, and Glu116. Metoserpate also engaged in a pi-alkyl interaction with Tyr115 and Pro117, as well as two cation-pi interactions with Glu104 and Glu116. These interactions played a crucial role in the stability and specificity of the complex (Figure 6). Hydrophobic contact area was also established between metoserpate and the binding site AA residues Lys98 and Tyr115.

In contrast, the bond network analysis conducted prior to the MD simulation revealed specific interactions between metoserpate and TNF*α*, including a cH-bond network with Gln102, a pi-alkyl interaction with Arg103, and cation-pi interactions with Glu104. However, the subsequent MD simulation analysis yielded intriguing findings, indicating an enhanced binding affinity and selectivity of metoserpate towards TNF*α*. This improvement in binding was accompanied by the generation of more favourable and specific interactions.

These results are further supported by the observed flexibility in the root mean square fluctuation (RMSF) output of the TNF*α* and metoserpate complex. The MD simulations have provided valuable insights into the intricate molecular interactions between the TNF*α* and metoserpate, unravelling the complexities of protein-ligand complexes.

By elucidating the dynamic behaviour and uncovering the structural changes that occur during the simulation, the MD simulations offer a deeper understanding of the binding mechanism and contribute to the overall comprehension of the interactions between TNF*α* and metoserpate.

#### 3.6.3. Overall Bond Network Assessment

The Hyde scoring method has proven to be a valuable computational tool in drug discovery for estimating the binding affinity between a protein and a ligand, utilizing their interaction energy [94]. In the present study, we employed the Hyde score assessment method to evaluate the binding affinity of two ligands, metoserpate and *yohimbine*, with the protein TNF*α*, with a specific focus on the amino acid residues within the binding site (Table 2).

The study's findings revealed that metoserpate exhibited a slightly lower Hyde score (-1.1 kJ/mol) in comparison to *yohimbine* (-1.0 kJ/mol and 0.2 kJ/mol) when interacting with Gln102 (Table 2). This indicates that metoserpate possesses a marginally better binding affinity with TNF*α* when compared to *yohimbine*, although the difference observed is relatively small. These results shed light on the relative strengths of the interactions between metoserpate and TNF*α*, providing insights into the binding affinity. This information contributes to the understanding of the potential efficacy of metoserpate as a potential therapeutic agent targeting TNF*α* in the context of AD management.

The post-MD simulation analysis revealed the involvement of amino acid Lys98 in the conventional hydrogen bond formation, as well as the formation of a nonconventional hydrogen bond network with Pro113, which was originally not part of the binding site AA residues (Figure 6). This post-MD simulation analysis generated more bond diversity, and bond number compared to the TNF*α*-*yohimbine* and TNF*α*-metoserpate complexes. This demonstrates the importance of post-MD simulation analysis in providing a more comprehensive understanding of protein-ligand interactions beyond what can be predicted through initial scoring methods alone.

The findings of this study also suggest that the binding affinity of a ligand with a protein may be influenced by amino acid residues outside of the initial binding site. This is consistent with previous studies that have shown the importance of protein flexibility and dynamics in ligand binding [95]. It is possible that the nonconventional hydrogen bond network identified in the post-MD simulation analysis plays a critical role in the binding affinity between TNF*α* and metoserpate.

## 4. Conclusion

In summary, our study employed the Hyde score assessment method to evaluate the binding affinity of metoserpate and *yohimbine* with TNF*α*, with a specific focus on the binding site amino acid residues. While metoserpate generated a lower Hyde score than *yohimbine* with the key binding site amino acid Gln102, further investigation using postmolecular dynamics (MD) simulation analysis demonstrated the involvement of additional amino acid residues in the binding affinity. The results indicated that metoserpate has the potential to inhibit TNF*α* and thus presents as a promising candidate for further study as a therapeutic agent for TNF*α*-related diseases. Additionally, our work showcases the utility of *yohimbine* as a query compound to identify structurally similar drugs from the DrugBank database in the context of drug repurposing. Specifically, our study identified metoserpate as a potential inhibitor of TNF*α* using a computational approach that combined molecular docking and MD simulation. This approach allowed for a more comprehensive and nuanced understanding of the binding affinity of metoserpate with TNF*α* and provided insights into the potential mechanisms of inhibition. Furthermore, our use of *yohimbine* as a query compound helped identify metoserpate as a structurally similar compound with potential therapeutic properties. Overall, these findings represent a significant step forward in the development of metoserpate as a potential therapeutic agent for TNF*α*-related diseases. However, further research is needed to validate these findings through *in vitro* and *in vivo* (in a physiologically relevant cell line, fly models, and/or animal models) studies and to optimize the efficacy of metoserpate as a drug candidate.

## Figures and Tables

**Table 1:** Pharmacokinetic properties of *yohimbine.*

GI absorption	BBB permeant	Lipinski	Ghose	Veber	Egan	Muegge
High	Permeant	Yes	Yes	Yes	Yes	Yes

Permeant = blood-brain barrier permeant; Yes = no violation; BBB = blood-brain barrier.

**Table 2:** Hyde's score estimates and TNF*α* binding site amino acid residues.

Compound	Hyde score (kJ/mol)	Bond interaction (ligand ➔ protein)	Binding site amino acid residues
*Yohimbine*	-1.0 0.2	N5 ➔ Gln102 O3 ➔ Gln102	Cys69, Lys98, Ser99, Pro100, Cys101, Gln102, Arg103, Glu104, Thr105, Trp114, Tyr115, Glu116, and Pro117
Metoserpate	-1.1	N8 ➔ Gln102	

Gln102 = the key amino acid residue involved in the hydrogen bond formation.

**Table 3:** Drugs that are 75% or more structurally similar to *yohimbine.*

Drug (ID)	Status	% similarity	Chemical formula
Metoserpate (DB11530)	Vet approved	0.992	C_24_H_32_N_2_O_5_
Deserpidine (DB01089)	Approved	0.960	C_32_H_38_N_2_O_8_
18-Methoxycoronaridine (DB15096)	Investigational	0.942	C_22_H_28_N_2_O_3_
CP-320626 (DB03383)	Experimental	0.764	C_23_H_23_C_l_FN_3_O_3_
Rescinnamine (DB01180)	Approved	0.823	C_35_H_42_N_2_O_9_
Reserpine (DB00206)	Approved, investigational, withdrawn	0.809	C_33_H_40_N_2_O_9_
Raubasine (DB15949)	Experimental	0.873	C_21_H_24_N_2_O_3_
Methoserpidine (DB13631)	Experimental	0.812	C_33_H_40_N_2_O_9_
(7as,12ar,12bs)-1,2,3,4,7a,12,12a,12b-Octahydroindolo[2,3-a]Quinolizin-7(6h)-One (DB02191)	Experimental	0.767	C_15_H_16_N_2_O
Vinburnine (DB13793)	Experimental	0.751	C_19_H_22_N_2_O

Status = FDA approval status; Drug ID = DrugBank ID.

**Table 4:** Pharmacokinetic properties of the 10 DrugBank compounds.

Drug	BBB permeant	GI absorption
Metoserpate (DB11530)	Yes	High
Deserpidine (DB01089)	No	High
18-Methoxycoronaridine (DB15096)	Yes	High
CP-320626 (DB03383)	No	High
Rescinnamine (DB01180)	No	High
Reserpine (DB00206)	No	High
Raubasine (DB15949)	Yes	High
Methoserpidine (DB13631)	No	High
(7as,12ar,12bs)-1,2,3,4,7a,12,12a,12b-Octahydroindolo[2,3-a]Quinolizin-7(6h)-One (DB02191)	Yes	High
Vinburnine (DB13793)	Yes	High

BBB = blood brain-barrier; GI = gastrointestinal.

## Data Availability

The PDB file was obtained from the RCSB Protein Data Bank (http://www.rcsb.org/). The 3D conformer structure of *yohimbine* was obtained from the PubChem database (https://pubchem.ncbi.nlm.nih.gov/). The data generated in this research, including the utilized compounds, molecular docking outcomes, and molecular dynamics simulation data, are accessible upon request to the corresponding author.
